# The Fading Wild Plant Food–Medicines in Upper Chitral, NW Pakistan

**DOI:** 10.3390/foods10102494

**Published:** 2021-10-18

**Authors:** Muhammad Abdul Aziz, Zahid Ullah, Muhammad Adnan, Renata Sõukand, Andrea Pieroni

**Affiliations:** 1 University of Gastronomic Sciences, Piazza Vittorio Emanuele II 9, 12042 Pollenzo, Italy; a.pieroni@unisg.it; 2 Center for Plant Sciences and Biodiversity, University of Swat, Kanju 19201, Pakistan; zahidtaxon@uswat.edu.pk; 3 Department of Botanical and Environmental Sciences, Kohat University of Science and Technology, Kohat 26000, Pakistan; ghurzang@hotmail.com; 4 Department of Environmental Sciences, Informatics and Statistics, Ca’ Foscari University of Venice, Via Torino 155, 30172 Venezia, Italy; renata.soukand@unive.it; 5 Department of Medical Analysis, Tishk International University, Erbil 4401, Iraq

**Keywords:** food–medicines, Mastuj, Chitral, WFPs, pharmacological properties

## Abstract

The subject of food–medicines (foods ingested in order to obtain a therapeutic activity or to prevent diseases) is garnering increasing attention from both ethnobiologists and ethnopharmacologists as diet-related chronic diseases are one of the major problems resulting in a large proportion of deaths globally, which calls for interest from the scientific community to make sensible decisions in the field of food and medicine. In this regard, the current study is an important attempt at providing baseline data for developing healthy and curative food ingredients. This study aimed at recording the culinary and medicinal uses of wild food plants (WFPs) in the remote Mastuj Valley, located at the extreme north of Chitral District, Pakistan. An ethnobotanical survey was completed via 30 in-depth semi-structured interviews with local knowledge holders to record the food and medicinal uses of WFPs in the study area. A total of 43 WFPs were recorded, most of which were used as cooked vegetables and raw snacks. Leaves were the most frequently used plant part. A remarkable proportion (81%) of use reports for the recorded wild plant taxa were quoted as food–medicines or medicinal foods, while very few were reported as either food or medicines, without any relationship between uses in these two domains. Previous ethnomedicinal studies from nearby regions have shown that most of the recorded wild plants have been used as medicines, thus supporting the findings of the current study. A literature survey revealed that many of the reported medicinal uses (33%) for the quoted WFPs were not verifiable on PubMed as they have not been studied for their respective medicinal actions. We observed that most of the plants quoted here have disappeared from the traditional food and medicinal system, which may be attributed to the invasion of the food market and the prevalence of allopathic medicine. However, knowledge of these wild plants is still alive in memory, and women are the main holders of cultural knowledge as they use it to manage the cooking and processing of WFPs. Therefore, in this context, we strongly recommend the preservation of local biocultural heritage, promoted through future development and educational programs, which could represent a timely response to the loss of cultural and traditional knowledge.

## 1. Introduction

The famous saying “Let food be thy medicine and medicine be thy food” by Hippocrates is finding an important place in today’s world full of diet-related chronic diseases. The Greeks were not the first humans to focus on the medicinal value of food as there had been other traditional medical systems, including Ayurvedic and Traditional Chinese Medicine, that promoted the idea of food being medicine at the same time [1]. Diet has remained an important factor in preventing certain diseases and it always played a pivotal role in the early history of many Western biomedicines [2]. A perfect diet is a powerful tool in maintaining a healthy life [3], but it is a misfortune that the prevalence of an unhealthy diet has given rise to serious chronic diseases in people of the so-called modern world [4]. We are living in “*a time of crisis*” where one fifth of all deaths are attributed to a suboptimal diet [5]. In this widespread situation, individual interactions with the healthcare system are an important opportunity to provide a set of evidence-based food and nutrition interventions [6]. Research has shown that these interventions introduced into the healthcare system might be linked to improved health outcomes and reduced healthcare costs and usage [7,8,9]. It is believed that the close integration of nutrition interventions and food could provide a solid foundation for the initiative to include medically tailored meals, integrating nutritional strategies into healthcare systems, which is generally referred to as “*food is medicine*” [6].

Wild food plants (WFPs) have played an important role in feeding humans for millennia, and they are considered an alternative source of healthy and nutritious food [10,11]. The literature indicates that nearly 7000 wild species are recognized as edible [12] and many of them have proven medicinal benefits, producing positive health outcomes, thus creating a solid foundation for generating novel functional foods [13]. Shikov et al. [13], referring to Gammerman and Grom (1976), stated that WFPs have been selected not only for their good taste or smell, but also for their important pharmacological potential [14]. Only a very small percentage of WFPs are used on a large scale, while the majority of them are still overlooked by mainstream society [13]. Wild plants, due to their phyto-constituents, retain remarkable medicinal, pharmacological, and nutraceutical potential to cure diseases, and thus Lim [12] asserted that herbal infusions can add small quantities of essential micronutrients and vitamins to daily intake to help restore balance among nutrients and maintain health.

There are hundreds of field ethnobotanical studies that have been devoted to either food uses or medicinal uses of wild species, but to date the subject of food–medicines in cultural contexts is poorly researched even though studies have shown a general resurgence of interest in wild food plants for their nutritional and medicinal values so as to broaden the diversity of the human diet [15,16]. Kunakova et al. [17] argued that knowledge on the positive pharmacological potential of WFPs could provide a better opportunity to integrate these ingredients into the modern pharmacopeia to develop functional products with useful benefits.

North Pakistan is making important socio-ecological space for traditional ecological knowledge of wild food and medicinal resources. Several ethno-medicinal studies have been carried out in the region over the last decade [18,19,20,21]. More recently, a body of literature on wild food plant knowledge has also emerged [22,23]. Nevertheless, the subject of food–medicines is a relatively new subject in local and cultural contexts, as in many other parts of the world. The ethnobotanical literature has shown that the cultural knowledge of food–medicines retained by local communities in the region could provide an effective platform for devising innovative strategies and policy inferences in future healthcare interventions. Therefore, the overarching aim of this study was to document WFPs along with their folk medicinal uses in order to produce baseline data for further scientific discoveries related to food-based medicine.

The current study was undertaken in Mastuj Valley, Chitral District, North Pakistan. It is a remote mountain valley that has never been investigated with regard to WFPs and other wild food resources. This is the first study to record the biocultural food heritage of WFPs along with their medicinal importance. The specific objectives of this study were:

1. To document the culinary and medicinal uses of WFPs gathered among the local communities;
2. To cross-check the findings of the current study with previous ethnobotanical studies conducted in nearby regions and to research the ethnopharmacological relevance of the recorded plant taxa.

The current study represents the very first attempt at gathering an important store of information on local biocultural heritage from the remote Mastuj Valley focusing on wild food plants and their perceived medicinal properties.

## 2. Materials and Methods

### 2.1. Study Area and Studied Community

**Figure 1:**
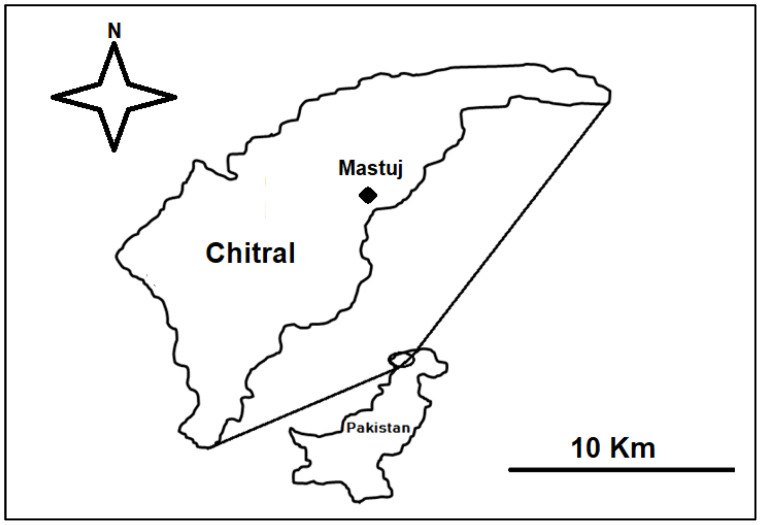
Location of the study area in Pakistan.

The study area is part of the Hindukush Mountain range and is located at the extreme north of Chitral District (Figure 1). The area is populated by the Kho people, who speak Khowar, the dominant linguistic group in Chitral. The Kho community has been living in the area for centuries. They are horticulturists that also rear livestock, and many individuals earn their livelihood from diverse types of off-farm activities. They have a long-term, inextricable link with nature and local resources, and they have amassed a remarkable store of traditional ecological and cultural knowledge on the local ecology and environment.

### 2.2. Field Study

An ethnobotanical survey was carried out in the month of June 2021 at different study sites across the valley. The main purpose of the study was to record the food and medicinal uses of wild plant taxa, commonly known as WFPs, and mushrooms among the local communities. A total of 30 people, with deep knowledge of and longtime experience with WFPs, were interviewed for the study. The interviewees were selected among middle-aged and elderly individuals (range: 50 to 72 years old), including local farmers and shepherds, as they retain sufficient knowledge of local wild food and medicinal resources. We only conducted interviews with men as there were socio-cultural constraints regarding the interviewing of women in the study area. Prior to each interview, we obtained verbal consent from each participant to record and publish their knowledge. The interviews were conducted in the Urdu language, and we employed the services of a local translator to avoid any difficulty in communicating with the participants. Throughout the study, recommendations given by the International Society of Ethnobiology were strictly followed [24]. The detailed interviews with study participants focused on gathered WFPs, i.e., plants used as cooked vegetables, snacks, recreational teas, and seasoning. We also inquired about the uses of WFPs in lacto-fermentation processes and dairy products. After detailed discussions on food uses, we focused on documenting the medicinal uses of the recorded taxa. We recorded the local names of each taxon in the Kho language, which were revised multiple times, by asking different elderly individuals to avoid any errors in local nomenclature. Qualitative ethnographic data were obtained through direct observations and in some cases through open-ended questions. At the end of the survey, recorded plant taxa were gathered, and the collected specimens were identified by the third author with the help of the *Flora of Pakistan* [25,26,27,28]. The specimens were assigned voucher numbers and incorporated into herbaria, subsequently submitted to the Department of Botany, University of Swat, Khyber Pakhtunkhwa, Pakistan. The scientific nomenclature of each taxon was verified through The Plant List database [29] and family assignments were consistent with the Angiosperm Phylogeny Website [30]. The Index Fungorum [31] was utilized to cross-check fungal nomenclature. The study was approved by the Ethics Committee of the University of Gastronomic Sciences, Pollenzo, Italy.

### 2.3. Data Analysis

The gathered data were entered into an MS Excel spreadsheet. The recorded WFPs were categorized into three groups based on folk uses, which were then visually displayed in a proportional Venn diagram drawn using free software (http://bioinformatics.psb.ugent.be/webtools/Venn/, accessed on 18 August 2021). We also compared our newly obtained data with the existing ethnobotanical literature of Pakistan to cross-check the quoted food and medicinal uses of the recorded WFPs with previous field studies. Moreover, we also researched the pharmacological potential of the recorded WFPs via peer-reviewed scientific literature on PubMed.

## 3. Results

### 3.1. Wild Food Plants and Their Uses

The use of a total of 43 taxa belonging to 25 families was recorded (Table 1). The WFPs most frequently quoted as wild vegetables were *Amaranthus hybridus*, *Capparis spinosa*, *Chenopodium album*, *Eremurus stenophyllus*, *Ferula hindukushensis*, *Portulaca quadrifida*, and *Rumex dentatus.* The majority of plants, which were largely harvested in their early growth stages, were used as cooked vegetables (24 taxa, e.g., *Amaranthus hybridus*, *Capparis spinosa*, *Chenopodium album*, *Eremurus stenophyllus*, *Portulaca quadrifida*) and consumed as raw snacks (15 taxa, e.g., *Berberis parkeriana*, *Chenopodium foliosum*, *Cotoneaster nummularius*, *Crataegus songarica*, *Elaeagnus angustifolia*). Among the reported plants, a few were cited for making teas. Leaves (22 taxa) were frequently used in culinary preparations.

**Figure 2:**
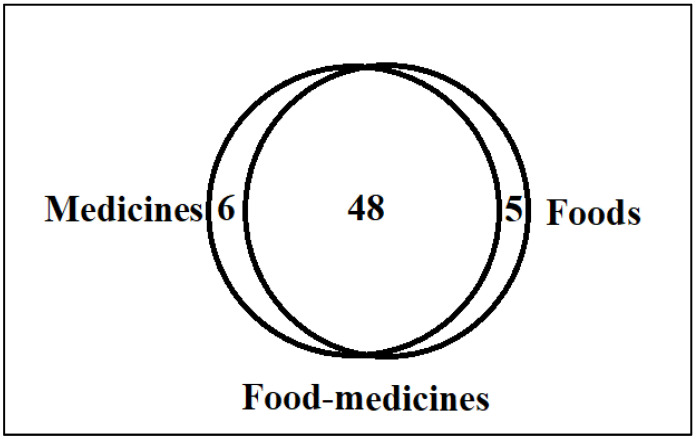
Venn diagram showing the different uses of locally gathered WFPs in the study area.

We documented a total of 50 food uses for the recorded WFPs in which 35 use reports comprise 70% of all food uses, and these uses were mentioned by more than half of the informants, indicating a large store of knowledge still retained by the local inhabitants. Besides the food uses recorded for the 43 WFP taxa, 42 taxa were also mentioned by the study participants as either functional food or medicinal food, which we categorized into three groups (Figure 2) purely on the basis of the processing and preparation of these wild ingredients. We observed a huge overlap of use reports that were commonly cited as useful both for food and medicine, i.e., plants were consumed as food to obtain the target medicinal action, and these taxa are known as *food–medicines* or *medicinal foods*. The use reports that did not overlap represent divergences in the form of their processing and preparation, i.e., without any relationships between uses in these two domains for any targeted health actions. Wild food plants were mostly used to treat digestive issues (16 taxa, e.g., *Amaranthus hybridus*, *Carum carvi*, *Convolvulus arvensis*, *Ferula hindukushensis*, *Lepyrodiclis holosteoides*, and *Mentha longifolia*), followed by cardiovascular problems (four taxa, e.g., *Ferula hindukushensis*) and increased blood (four taxa, e.g., *Crataegus songarica*). Of the quoted taxa, nine plants were used or considered good for more than one ailment, i.e., *Berberis parkeriana*, *Capparis spinosa*, *Elaeagnus rhamnoides*, *Ferula hindukushensis*, *Medicago sativa*, *Mentha longifolia*, *Nasturtium officinale*, *Rheum ribes*, and *Rumex dentatus.* Moreover, 29 taxa were used for a single health problem. The most common health issue among gastric problems was constipation, which was frequently treated with green vegetables including *Amaranthus hybridus*, *Capsella bursa-pastoris*, *Chenopodium album*, *Chenopodium murale*, and *Malva neglecta.*

The link between disease and food has been widely recognized as the foundation of preventive nutrition. Over the last few decades, there have been other examples in Europe of wild plant species that have been introduced from other continents, such as Africa, e.g., *Aspalathus linearis* (Burm.f.) R. Dahlgren and to a lesser extent *Cyclopia* sp. or *Hoodia gordonii* (Masson) Sweet ex Decne., to stimulate appetite. Similarly, other exotic fruits rich in vitamin C, such as *Malpighia glabra* L. from South America, are now marketed as food supplements [88]. We also recorded four taxa that were considered as healthy foods without targeting any particular health problem. Elaborating on the concept of healthy food, a 70-year-old man explained:

“In the past we gathered WFPs from the mountains and consumed them as these plants gave us more power and energy. We were healthy, more capable of working hard and climbing mountains. But now with the arrival of bazaars and local markets, everyone can find *Bazaari* vegetables and people don’t bother to gather these WFPs from the mountains, except for a few individuals that graze their animals or hunt. WFPs are more powerful than cultivated ones and old people prefer these much more”.

Wild food plants have been recognized as “depurative” and they are a kind of “folk functional food” as defined by Pruess [89]; that is, food that has nutritional value or is eaten for pleasure but which also has beneficial effects on health. We also carried out a literature survey to determine the medicinal uses of these taxa and found a total of 27 food taxa that were reported as *food-medicines* in nearby regions (Table 1). Similarly, the literature also revealed 13 taxa among the recorded plants that were used both in the food and medicinal domains without any relationship among their respective uses. Looking at the overall results, we can presume that many plants were utilized in a multifunctional way, forming a complex web of relationships as presented in Table 1.

**Figure 3:**
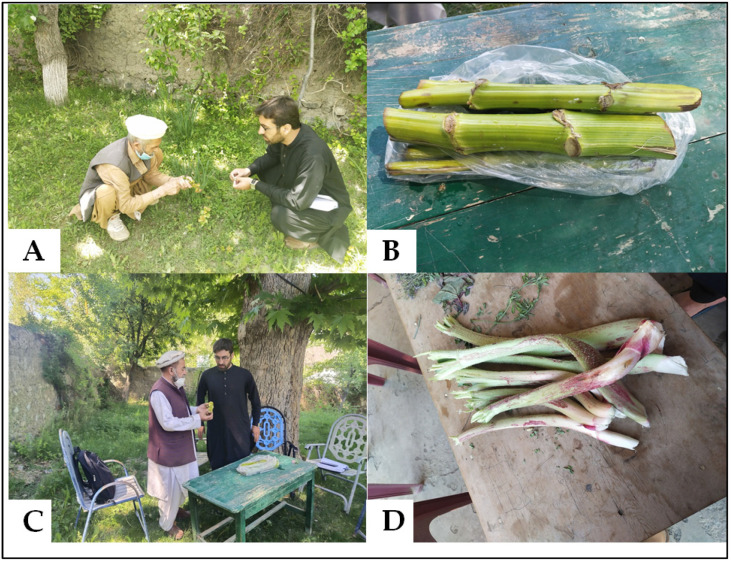
Plants found in some homes in the study area; (**A**,**C**) local informants explaining the uses of WFPs (**B**) *Ferula hindukushensis* and (**D**) *Rheum ribes*.

We observed that medicinal knowledge was popular among very few participants while culinary uses of the recorded taxa were widely known in the local communities, especially among elderly individuals. In the area, both men and women were involved in the gathering of WFPs: men generally collected the WFPs in the mountains while women handled the processing and cooking. Therefore, both men and women have deep cultural knowledge of these natural resources. During the study, we were not allowed to interview women, but we observed that sometimes male informants asked their wives to help recall some information related to WFPs. As the cooking is performed by women, they may have a deeper attachment to, and thus stronger memories of, WFPs compared to men. In the study area, during the last twenty years, the dependency of local communities on WFPs has decreased tremendously due to the invasion of local food markets and the prevalence of allopathic medicines. However, we encountered people who are still practicing pastoralism and hunting, engaged in the gathering and consumption of WFPs. It is worth mentioning that the rapid economic evolution and change in social life of the studied community have significantly interrupted the intergenerational transmission of traditional knowledge, although middle-aged and elderly individuals possess abundant cultural knowledge of the local resources. We observed in some homes some important WFPs (Figure 3) that were gathered from pastures located at higher elevations in the mountains as they were considered useful for their medicinal properties. We were told that younger people, who tend to live in cities, were not familiar with the food value of most WFPs, and they are more interested in receiving a modern education.

The wild vegetables that have nearly disappeared from the local food system include *Capsella bursa-pastoris, Convolvulus arvensis, Descurainia Sophia, Lepyrodiclis holosteoides, Malva neglecta, Medicago sativa, Plantago lanceolata, Portulaca quadrifida, Rumex dentatus, Silene conoidea*, and *Taraxacum campylodes*. The disappearance of these taxa is linked not only to globalization and social change but also to environmental change. Studies have shown that environmental change in the Hindukush and Himalayan region has greatly impacted the local natural resources. Measuring the LEK in West Pakistan, Abbas et al. [90] reported that the cultural knowledge of WFPs was partially eroded as they found that the majority of quoted plants were mentioned by only one third of the participants. However, the TEK of WFPs is still alive in both the memory and current practices of the local inhabitants; for example, communities living around Takht-e-Sulaiman Hills, NW Pakistan [91], as well as the Hindu Kush mountains in North Pakistan [23], have retained important traditional knowledge of WFPs. The popularity of WFPs as food–medicines has also been recognized in various communities living in North Pakistan. In a nearby region, Aziz et al. [22] identified some important plant species used in both food and medicine, for instance, *Allium carolinianum* was generally preferred by elderly individuals for treating joint pain. *Capparis spinosa* was considered an important economic plant gathered by the local communities and is well known for treating liver problems, as well as diabetes, hepatitis, cough, cold, fever, and malaria. Shi et al. [92] reported that in China, Korea, Japan, and other countries of Southeast Asia, medicinal plants are frequently used in both everyday foods and functional foods for medical purposes. Heinrich [93] states that the blurring of the food and medicine interface is a common theme across multiple contexts and cultures.

### 3.2. Pharmacological Effects of the WFPs Recorded in the Study

Research has shown that wild plant species have enormous capacity for certain pharmacological effects. The question of the continuum between food and medicine is of great interest to (ethno-)pharmacologists [94,95,96,97,98]. Pieroni et al. [99], for instance, reported that non-cultivated food species have strong antioxidant potential. Looking at the pharmacological profile of the quoted WFPs, we found that a sufficient proportion of WFPs were not verifiable on PubMed, i.e., 22 medicinal use reports out of 54 use reports had some pharmacological properties that have already been studied. During the literature survey, we found twenty-five in vivo and twenty-four in vivo studies. Among the quoted plants, 12 taxa (*Amaranthus hybridus*, *Capsella bursa-pastoris*, *Carum carvi*, *Chenopodium album*, *Convolvulus arvensis*, *Crataegus songarica*, *Malva neglecta*, *Medicago sativa*, *Mentha longifolia*, *Nasturtium officinale*, *Taraxacum campylodes*, and *Thymus linearis*) have proven antioxidant potential (Table 1). These plant taxa were used to treat gastric and cardiovascular issues, for which antioxidant agents play a crucial role in the prevention of such health problems. Antioxidants have the potential to scavenge free radicals inside the human body, which is beneficial as the presence of free radicals can have injurious effects and cause serious ailments, such as inflammatory cardiovascular diseases, cancer, and cataracts [100]. Plants are considered an important source of natural antioxidants [101], while synthetic antioxidants have been reported to have serious physiological effects on the human body, and therefore scientists have emphasized that plant-derived natural antioxidants are important alternatives to synthetic ones [102]. In recent times, a notable trend has emerged in which dietary plants are utilized as therapeutic antioxidants to decrease the rate of chronic diseases across the globe [101]. It has been reported that there is an inverse relationship between the dietary intake of antioxidant-rich foods and medicinal plants and the incidence of human diseases. It is believed that natural antioxidants are much cheaper than synthetic ones and have no harmful effects on the human body. Many plants have been investigated for their antioxidant potential including potatoes, tomatoes, spinach, legumes, and a variety of vegetables [103], and many fruits have been found to have strong antioxidant potential, such as cherries, berries, citrus fruits, olives, and prunes. Similarly, black and green teas have been extensively studied for their antioxidant activity since they contain up to 30% phenolic compounds by dry weight [104,105]. This is the reason dietary intervention studies have been devoted to assessing the impacts of increasing consumption of antioxidant foods on cardiovascular disease risk [106]. It is equally important to isolate the various phytochemicals present in these different food and medicinal species so as to make them part of important food supplements that would have more positive impacts than synthetic food products. Natural antioxidants, particularly carotenoids and polyphenols, display a wide range of biological effects, including anti-aging, anti-inflammatory, anticancer, and anti-atherosclerosis, and thus the careful extraction and proper assessment of antioxidants from both the recorded food and medicinal plants are essential for exploring potential antioxidant sources and promoting their applications in functional foods, pharmaceuticals, and food additives [107]. In today’s world, the exploration of novel food supplements is crucial and, in this regard, systematic investigations of such plants, also known as food-medicinal plants, are providing an important opportunity, especially in isolated areas.

### 3.3. Inclusion of LEK of WFPs with Medicinal Properties in Educational Platforms

During this study, we observed that younger generations have become detached from the environment supporting the learning of LEK as they are more interested in obtaining a modern education. It is important to note that the rapid social change caused by modernization and globalization processes has made it imperative to revitalize cultural and traditional knowledge through proper educational programs. However, the revitalization of LEK in educational programs is a two-way street involving both relevant stakeholders as well as local communities to effectively transmit cultural knowledge of local ecological resources. We strongly believe that bringing LEK knowledge into actual classroom practices will not only promote biocultural heritage, but it will also help students to understand science-based local ecological experiences. Curriculum designers and relevant policy makers should pay serious attention to the erosion of LEK and try to involve ethnobiologists in designing a culturally ecologically responsive curriculum to give LEK and the local biocultural heritage of WFPs the chance to survive and evolve along with modern scientific knowledge. Proper incentives should be given to local knowledge holders in order to secure their services. It is strongly recommended that policy makers pay serious attention to the efficient revitalization, through educational programs, of the LEK that has been an important cultural element of local ecological communities for generations. It should be kept in mind that LEK plays an important role in managing the local environment and resources, and therefore the popularization of WFPs with health benefit properties could be a better option in the modern world. We suggest that LEK should be part of policy frameworks in future development programs because traditional knowledge has played a crucial role in organizing both local ecosystems and local subsistence economies. Similarly, medicinal plants have supported the healthcare system for centuries, and thus policy makers should involve ethnobiologists around the country to formulate better strategies to protect and practically revive LEK and local biocultural heritage through educational and development programs to achieve sustainable development goals [108].

## 4. Conclusions

The current study reported an important store of cultural knowledge of WFPs used as food–medicines among local communities residing in a remote valley of Chitral, North Pakistan. Most of the plants quoted in this study that were processed for culinary uses were also used for their perceived health benefits as they are commonly recognized as *medicinal foods* or *food-medicines*. Previous ethnobotanical studies conducted in nearby regions have also shown that most of the recorded food plant taxa have important medicinal uses for the local communities, thus strengthening and supporting the findings of the current research. A literature survey also indicated that about one third of the quoted plants have not been tested for their pharmacological potential and were not verifiable on PubMed as they have not been studied for their respective medicinal actions. It is important to note that biocultural heritage is gradually eroding due to rapid social change and urbanization processes. On the basis of the findings of the current study, we recommend verifying, through standard laboratory techniques, the pharmacological potential of WFPs for the preparation of better food and nutrition supplements to help combat some human diseases. We also emphasize the revitalization of WFP-centered local biocultural heritage through future development and educational programs, which could represent a timely response to the loss of LEK. Lastly, we also suggest that future research should focus on the traditional ethnobotanical knowledge held by women in the study area.

## Figures and Tables

**Table 1:** WFPs used as food–medicines, along with their pharmacological properties, reported among local communities living in North Pakistan.

Botanical Taxon; Family; Botanical Voucher Specimen Code	Recorded Local Name	Parts Used	Recorded Local Food Uses	Perceived Medicinal Uses	Medicinal Uses Recorded in Nearby Regions, NW Pakistan	Pharmacological Potential
*Allium carolinianum* DC.; Amaryllidaceae; SWAT005988	Latruk	Leaves	Cooked ***	Hepatitis **	Joint pain [22].	In vivo: immunomodulatory potential [32].
*Amaranthus hybridus* L.; Amaranthaceae; SWAT005470	Krui shakh	Leaves	Cooked ***	Constipation ***	Vermicide, tonic [33]. Young leaves are used as vegetables, which are a laxative [34].	In vitro: antioxidant activity [35].
*Astragalus psilocantros* L.; Fabaceae; SWAT005995	Garmenzo	Stem	Internal part of stem: raw snack *	Internal soft part of the stem is put in the fire and then consumed to treat rheumatism *.	-	-
*Berberis parkeriana* C.K. Schneid.; Berberidaceae; SWAT005491	Choweng	Leaves, Fruit	Fruit: raw snack ***, Leaves: Chatni ***	The fruit is considered a blood purifier ***. A decoction of the root is used to reduce prolonged fever *.	The fruits are crushed, boiled in water, and then used to reduce fever [36]. Leaves and fruits are collected and juice is extracted and filtered, which can then be taken orally for the treatment of typhoid, jaundice, dyspepsia, blood purification, and muscular pain [34].	In vivo: anti-inflammatory and antioxidant activities. In animal models, berberine has neuroprotective and cardiovascular protective effects. In humans, its lipid-lowering and insulin-resistance improving actions have clearly been demonstrated in numerous randomized clinical trials [37]. In vitro: anti-oxidant [38], In vivo: anti-inflammatory [39], In vivo: anti-tumor [40], In vivo: anti-mutagenic [41], In vitro: anti-diabetic [38] properties of berberine.
*Capparis spinosa* L.; Capparaceae; SWAT005794	Kaveer	Fruit	Fruit: vegetable ***, Flower: extract is used in seasoning ***	Liver problems ***, typhoid *, and pneumonia *	The cooked vegetable is considered useful in treating liver problems, diabetes, hepatitis, cough, cold, fever, and malaria [22]. Floral buds are collected, dried, mashed with wheat flour, and cooked to prepare an aqueous extract called Kavirough, which is effective for the treatment of abdominal pain, malaria, and typhoid [34].	In vivo: anti-diabetic [42,43,44], In vivo: anti-hypertensive [45], In vitro: antimicrobial [46], In vivo: anti-inflammatory [47], In vivo: antihepatotoxic [48].
*Capsella bursa-pastoris* (L.) Medik.; Brassicaceae; SWAT005997	Palak	Aerial parts	Cooked ***	Constipation *	-	In vitro: antioxidant potential [49].
*Carum carvi* L.; Apiaceae; SWAT005966, SWAT005981	Hojooj	Fruit or seeds	Tea ***, seasoning ***, used in yogurt or dough and eaten to cool the brain *	Gastric problems ***	Seeds are boiled; herbal tea is made and is used for nausea and stomachache [34].	In vitro: antioxidant, anti-inflammatory potential [50]. In vitro: antimicrobial, anti-acetylcholinesterase, and antidiabetic [51].
*Chenopodium album* L.; Amaranthaceae; SWAT005499	Konakh	Leaves	Cooked ***	Constipation ***	Leaves are served as vegetables for bowel disorders, as a laxative for constipation [34].	In vivo: antioxidant and anti-inflammatory potential [52].
*Chenopodium foliosum* Asch.; Amaranthaceae; SWAT005510	Pilimarach	Fruit	Raw snack ***	Throat infection*	Juice is extracted from ripe and clean fruits and applied to the eye to treat infections. The ripe fruits are eaten raw for their taste; they are also used for eye infections [36].	-
*Chenopodium murale* L.; Amaranthaceae; SWAT000776	Dar konakh	Leaves	Cooked ***	Constipation ***	Especially used for abdominal pain, as a diuretic, and it is considered an anthelmintic [36].	-
*Cirsium arvense* (L.) Scop.; Asteraceae; SWAT000728	Chamcheer	Roots	Raw snack ***	-	-	-
*Convolvulus arvensis* L.; Convolvulaceae; SWAT005968; SWAT005966	Mishk	Leaves	Cooked ***	Leaves are cooked as vegetables and used to relieve constipation *.	Young leaves are used as vegetables to treat constipation [34]. The roots are dried, powdered, and used as a purgative, i.e., for evacuation of the bowels [36].	In vitro: antioxidant [53].
*Cotoneaster nummularius* Fisch. & C.A.Mey.; Rosaceae; SWAT005485	Mikeen	Fruit	Raw snack ***	Increase blood ***	The edible fruits are a blood purifier [34].	-
*Crataegus songarica* K. Koch; Rosaceae; SWAT005473	Goni	Fruit	Raw snack ***	Heart problems ***, increase blood ***	Fruits are edible and considered a cardio tonic [36].	In vivo: antioxidant effect and anticancer activity as it protects heart cells [54]. In vivo: hypotensive agent [55].
*Cucurbita pepo* L.; Cucurbitaceae; SWAT005999	Kado	Flowers	Cooked *	Cooling agent *	Vegetable is used to lower blood pressure [34].	-
*Descurainia sophia* (L.) Webb ex Prantl; Brassicaceae; SWAT005793, SWAT005513	Kheli kheli	Leaves	Cooked *	Brain tonic **	Young shoots and seeds are powdered and used for gas trouble and intestinal disorders. The decoction is used as apainkiller. Freshly collected leaves are consumed with milk for reducing high fever [36].	In vitro: anti-inflammatory, analgesic, and antipyretic effects, and antioxidant and anthelmintic activities [56].
*Elaeagnus angustifolia* L.; Elaeagnaceae; SWAT005806, SWAT005808	Sinjur	Fruit	Raw snack ***	Respiratory problems ***	Ripe fruits are boiled in water, sugar is added to enhance flavor, and a syrup is prepared. This syrup is used for sore throat and high fever [36]. Fruits are dried and powdered to treat asthma and cough. The gummy stem and branch resin is dried, then powdered, and used as a tonic shampoo for long, healthy, and silky hair [34].	-
*Elaeagnus rhamnoides* (L.) A.Nelson; Elaeagnaceae: SWAT005998	Mirghinz	Fruit	Raw snack *	Blood pressure *, asthma *, gastric problems *. The fruit is also used to treat abdominal pain **.	The juice obtained from the berries, called Buringogh, is used against high blood pressure and eye diseases. Berries are also applied to the face as a face mask to treat sunburn [34].	In vitro: cardiovascular and cerebrovascular protection, anti-tumor, anti-inflammatory, and anti-oxidation [57]. In vitro: antifungal, anti-psoriasis, anti-atopic dermatitis, and wound healing activities [58]. In vitro and in vivo: antioxidative and immunomodulating [59,60], In vivo: cardioprotective and antiatherogenic [60,61,62], In vitro: antibacterial and antiviral effects [63,64], In vitro and in vivo: healing effect on acute and chronic wounds [65,66,67], In vitro: antiradiation [68,69], In vitro: anti-inflammatory [70], In vitro: antidiabetic [71], In vitro and in vivo: anticarcinogenic [59,60], In vivo: hepatoprotective, and dermatological effects [72,73].
*Ephedra intermedia* Schrenk & C.A.Mey.; Ephedraceae; SWAT000772	Somani	Fruit	Raw snack *	The young shoots are ground and applied topically on the skin to treat stings and bites **.	Whole plant is boiled in water, crushed, and an aqueous extract is obtained, called Gholja in the Khowar language, which is used to treat facial sunburn, pneumonia, and gastric problems [34]. Ripe fruits are boiled in water and used for asthma and tuberculosis [36].	-
*Eremurus stenophyllus* (Boiss. & Buhse) Baker; Xanthorrhoeaceae; SWAT005967	Taikh shakh	Leaves	Cooked ***	Improve digestion **	-	-
*Fallopia dumetorum* (L.) Holub; Polygonaceae; SWAT005967, SWAT000774	Pindormishk	Leaves	Cooked ***	Healthy food **	Leaves are eaten to increase appetite, and used as a purgative, astringent, and diuretic [36].	-
*Ferula hindukushensis* Kitam.; Apiaceae; SWAT005983	Rawu	Stem, latex	Seasoning ***, pickle *** (along with vinegar)	Blood pressure *** and diabetes ***. The dried latex of the plant, locally known as *Hing*, is fastened around a child’s neck to prevent bad eyesight *. It is also used for piles ***. It is also used in some dishes to relieve flatulence and improve digestion ***.	Locally this species is used for cough, asthma, toothache, gastric problems, and constipation [36]. Young stems are cut resulting in the oozing out of a milky exudate. It is locally called *Hing*, which is used to treat stomachache, diabetes, and toothache [34].	-
*Foeniculum vulgare* Mill.; Apiaceae; SWAT006000	Bodioung	Seeds/Fruits	Seasoning ***	Tea: chest cough **	Seeds and fresh leaves are chewed for cough, abdominal pain, and pneumonia [34].	-
*Juglans regia* L.; Juglandaceae; SWAT006042	Birmough	Bark	Tea **	Healthy food *	Bark and leaves are used for gum and tooth diseases and for brightening teeth. A decoction of leaves is given for eczema. Seeds can be eaten to lower blood pressure [34].	In vivo: active against cardiovascular diseases [74].
*Juniperus excelsa* M.Bieb.; Cupressaceae; SWAT005497, SWAT005498	Sarwooz	Bark	Tea **	The ground fruit is given to children as a vermicide ***.	-	In vitro: antiparasitic, nematicidal [75].
*Lactuca serriola* L.; Asteraceae; SWAT005965	Chir jasho	Stem	Raw snack *	Healthy food *	-	-
*Lepyrodiclis holosteoides* (C.A. Mey.) Fenzl ex Fisch. and C.A. Mey.; Caryophyllaceae; SWAT000747	Birghal	Aerial parts	Cooked **	Laxative ***	-	-
*Malus domestica* Borkh.; Rosaceae; SWAT006043	Palough	Leaves	Cooked *	Healthy food **	-	-
*Malva neglecta* Wallr.; Malvaceae; SWAT006043	Suwachal Shakh	Leaves	Cooked ***	Constipation ***	Leaves are used as vegetables to treat constipation and other digestive problems, and they also act as a cooling agent [34].	In vitro: antioxidant potential [76].
*Medicago sativa* L.; Fabaceae; SWAT005797, SWAT005795	Moshish	Leaves	Cooked ***	Cooling agent *, appetizer *	-	In vitro: antioxidant effects [77].
*Mentha longifolia* (L.) L.; Lamiaceae; SWAT005792, SWAT005790	Ben	Leaves	Salad ***	Gastric problems ***. Root: vomiting **	Gastric problems. Herbal tea, made from the roots, called Benough, is used to cure fever, jaundice, and indigestion. The fresh and dried leaves are also eaten as a digestive and as a remedy for stomachache [34].	In vitro: antioxidant and antiemetic properties [78].
*Morchella esculenta* (L.) Pers.; Morchellaceae; SWAT004746	Kotchi	Aerial parts	Cooked ***	The extract is useful for treating eye problems *.	Useful for removing phlegm [33].	In vivo: anti-inflammatory effect [79].
*Nasturtium officinale* R.Br.; Brassicaceae; SWAT005482	Shiako shakh	Leaves	Cooked ***	Appetizer *, cooling agent ***, diuretic **	Respiratory problems [33]. Leaves serve as vegetables, which are used against dyspepsia and hepatitis [34].	In vitro: antioxidant activity [80].
*Plantago lanceolata* L.; Plantaginaceae; SWAT005962	Boeeko ligini	Leaves	Cooked **	Constipation ***	-	-
*Portulaca quadrifida* L.; Portulacaceae; SWAT005970	Pichili	Leaves	Cooked ***	Constipation ***	Joint pain [33].	-
*Rheum ribes* L.; Polygonaceae; SWAT004749	Ishpar	Leaf stalks	Raw snack *** Fermentation **	Appetizer *** and sexual tonic ***	Young stems and shoots are edible and eaten raw, which is used for treating flu and cough [34]. The root is used as a purgative. Leaf stalks are cooked as a vegetable. The unripe stem and leaf stalks are also eaten raw for their taste [36].	-
*Rosa webbiana* Wall. ex Royle; Rosaceae; SWAT005502	Throni	Fruit, Leaves	Fruit: raw snack ** Leaves: tea ***	Gastric problems **	A decoction is prepared from fruits boiled in water and then strained overnight to treat asthma [36]. The petals of these plants are collected, dried, crushed, and powdered; one teaspoon of this powder is added to tea to treat stomachache [34].	-
*Rubus fruticosus* G.N. Jones; Rosaceae; SWAT006044	Atchu	Fruit	Raw snack ***	Increase blood ***	Fruits are carminative and also used for diarrhea and intestinal looseness [34].	In vivo: antioxidant, anti-inflammatory, and gastroprotective properties [81]. In vitro: increase plasma catalase level [82].
*Rumex dentatus* L.; Polygonaceae; SWAT005468	Sirkonzur	Leaves	Cooked ***	Hepatitis *, increases blood **	Leaves are eaten to increase appetite; also used as a purgative, astringent, and diuretic [36]. The fresh leaves are collected, boiled, cut, and the resulting paste is mixed with tomato, onion, ginger, garlic, and salt (as required), and then fried in oil; this gravy is used as a laxative when eaten as a vegetable [34].	-
*Silene conoidea* L.; Caryophyllaceae; SWAT005481, SWAT005514	Hapupar	Aerial parts	Cooked ***	Paste from the aerial parts is made, which is used by girls to beautify their faces ***.	A paste is prepared from dried young leaves and seeds and then applied on pimples; also used to relieve backache [34]. A paste is prepared by grinding seeds and young leaves, which is then applied on pimples. This paste is also used for treating backache [36].	-
*Solanum americanum* Mill.; Solanaceae; SWAT005503, SWAT005803	Pilmilik	Fruit	Raw snack **	The berries are used by girls to heal sunburns ***.	Ripened fruits are collected to extract their juice, which is effective against eye irritation and sunburn. The fruits are eaten to alleviate stomachache [34].	In vivo: anti-inflammatory effect [83], anti-ulcerogenic and ulcer healing properties [84].
*Taraxacum campylodes* G.E.Haglund; Asteraceae; SWAT005972	Pawu	Leaves	Cooked ***	Constipation ***	Leaves and young shoots are served as vegetables to treat constipation, as well as liver and kidney disorders [34].	In vivo: hepatoprotective, anti-inflammatory, hypolipidemic, and hypoglycemic activities [85]. In vivo: antioxidant property preventing kidney damage [86]. In vivo: gastric emptying and smooth muscle motility [87].
*Thymus linearis* Benth.; Lamiaceae; SWAT000740	Sew	Aerial parts	Tea ***	Gastric problems ***	Tea is used for stomach disorders. It is also considered a carminative and tonic [36].	-

***: quoted by more than 50% of informants; **: quoted by between 50% and 25% of informants; *: quoted by less than 25% of informants.
